# Ancient Ephemeroptera–Collembola Symbiosis Fossilized in Amber Predicts Contemporary Phoretic Associations

**DOI:** 10.1371/journal.pone.0047651

**Published:** 2012-10-17

**Authors:** David Penney, Andrew McNeil, David I. Green, Robert S. Bradley, James E. Jepson, Philip J. Withers, Richard F. Preziosi

**Affiliations:** 1 Faculty of Life Sciences, The University of Manchester, Manchester, United Kingdom; 2 The Manchester X-Ray Imaging Facility, School of Materials, University of Manchester, Manchester, United Kingdom; 3 Department of Geology, Amgueddfa Cymru–National Museum Wales, Cathays Park, Cardiff, United Kingdom; 4 School of Earth, Atmospheric and Environmental Sciences, University of Manchester, Manchester, United Kingdom; Raymond M. Alf Museum of Paleontology, United States of America

## Abstract

X-ray computed tomography is used to identify a unique example of fossilized phoresy in 16 million-year-old Miocene Dominican amber involving a springtail being transported by a mayfly. It represents the first evidence (fossil or extant) of phoresy in adult Ephemeroptera and only the second record in Collembola (the first is also preserved in amber). This is the first record of Collembola using winged insects for dispersal. This fossil predicts the occurrence of similar behaviour in living springtails and helps explain the global distribution of Collembola today.

## Introduction

The number of species preserved in amber is staggering [1], but it is less common that behavioural interactions between species are preserved [2]. Rapid entombment and preservation in resin means that specimens can be preserved with life-like fidelity, sometimes even capturing the behavioural interactions between individuals. Phoresy is a form of behavioural symbiosis where an individual of one species is transported by another, larger species as a means of dispersal [3]. Examples of this behaviour in the amber fossil record usually relate to uropodid mites on other insects [4] or to pseudoscorpions attached by their pincers to the legs of wasps, flies or beetles [5]. Unusual examples of fossil phoresis include a mantispid larva on a spider [6], a histiostigmatid mite attached to a spider [7] and hyperphoretic mites attached to a phoretic pseudoscorpion being carried by a fly [8], all found in Eocene Baltic amber. None of the examples described to date are unusual based on our knowledge of the behaviour of their living relatives, but they are helpful for understanding when phoresy first evolved.

Phoresy in adult mayflies (Ephemeroptera) or springtails (Collembola) is unknown in living species. The only previously described fossil record of phoresy in either of these orders is that of five *Sminthurus longicornis* springtails aligned in a row and attached by hooking their antennae over the leg of a *Dicranopalpus ramiger* harvestman (Opiliones) in Eocene Baltic amber [9]. Given the nervous disposition of living springtails and their ability to jump away from danger using the furca (springing organ) on the underside of the abdomen, such preservation is indicative of extremely rapid entombment and almost instantaneous demise. It is presumably also for these reasons that phoretic behaviour on insects or other arthropods has not been noted in the extant springtail fauna. Collembola are distributed globally and readily colonize newly-formed islands. However, their modes of immigration are poorly understood and are thought to include dispersal as part of the aerial plankton [10] and via oceanic currents [11]. Indeed, recent studies focusing on collembolan biogeography did not consider phoresy as a potential dispersal mechanism in springtails [12], [13].

Mayflies are archaic hemimetabolous insects with stem group taxa that first appeared in the Carboniferous [14]. Their life history is highly unusual [15], most mayfly species spend about a year as an aquatic nymph. Adult mayflies live for a period that varies from less than one hour to a few days depending on the species. The primary function of the adult stage is reproduction and they are unable to feed. Upon maturation adult mayflies form mass mating swarms and tend not to use their power of flight for long-range dispersal. Indeed, mayfly dispersal is generally considered to be limited [16] to the same, or to proximate water bodies, with their global distribution today attributed primarily to vicariance. Therefore, mayflies would seem unlikely to be a good candidate to facilitate dispersal for phoretic organisms, although certain organisms hitch rides on their aquatic larvae [15], [17]. Nonetheless, recent molecular phylogenetic studies [18] have demonstrated that repeated long-range (including trans-oceanic) dispersal has occured in Ephemeroptera, and thus, it would appear that their dispersal abilities have been greatly underestimated [19].

In contrast to Baltic amber, where mayflies are reasonably common [20], they are very rare in Dominican amber [21], presumably indicating a lower availability of freshwater habitats in the Dominican amber forest. All four known Dominican Republic amber Ephemeroptera species belong in the family Leptophlebiidae: Atalophlebiinae [21], which is abundant in the Neotropical region today including the Greater Antilles [22].

**Figure 1:**
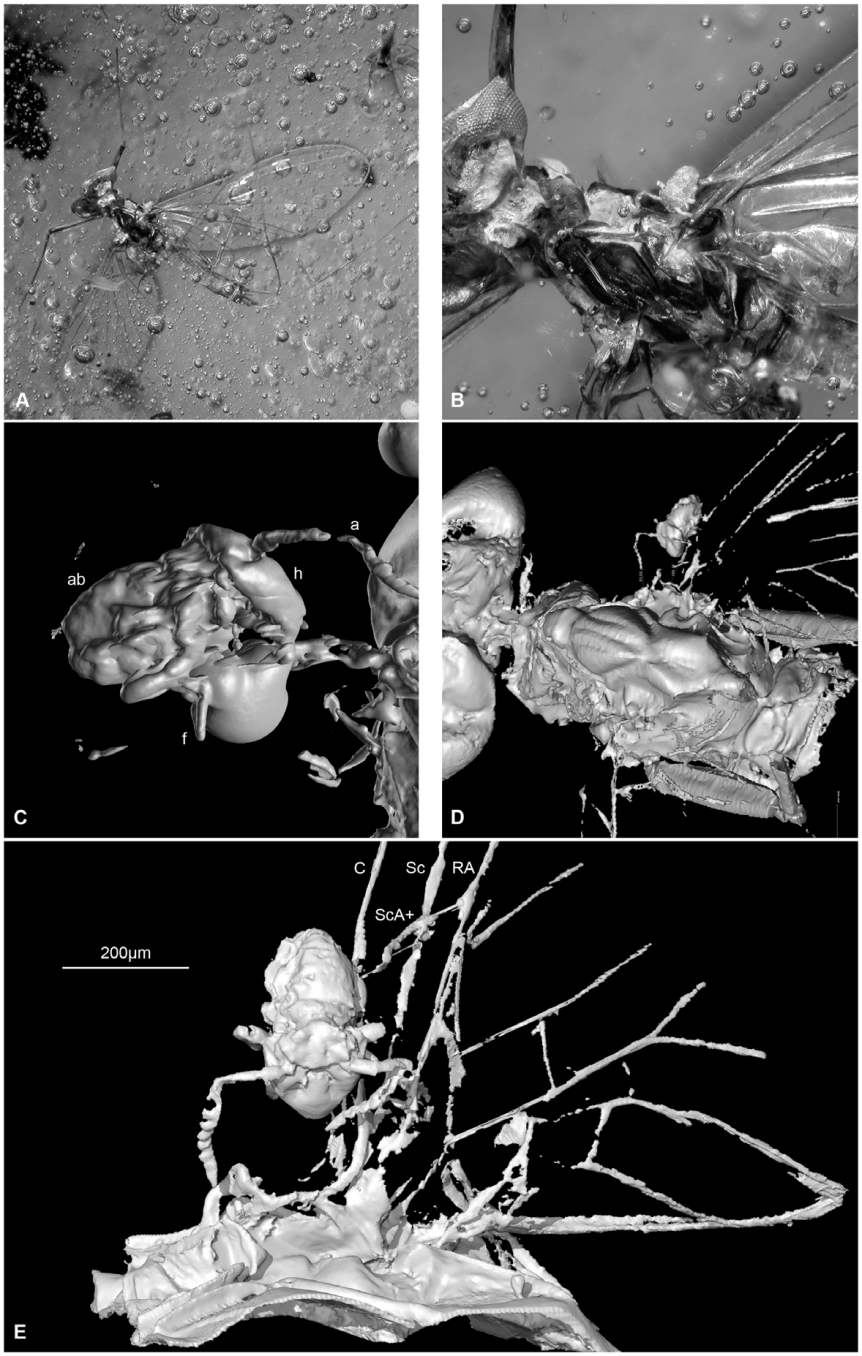
**Mayfly** ***Borinquena parva*** with a tiny phoretic Collembola syninclusion at the base of the right forewing; Miocene Dominican amber. A, photomicrograph of specimen in amber; B, close up of A; C, close up CT scan of collembolan in lateral view; D, CT scan of specimen – region as in B; E, CT scan of collembolan and mayfly wing in dorsal view; Abbreviations: a  =  antenna, ab  =  abdomen, f  =  furca, h  =  head; wing veins: C  =  costa, RA  =  radius anterior, Sc  =  subcosta, ScA+  =  costal brace. Body length of Collembola 228 µm.

Here we use X-ray computed tomography to identify a unique example of fossil phoresy in Dominican amber, involving a springtail and a mayfly.

## Materials and Methods

The amber originates from the La Bucara mine in the Cordillera Septentrional, Dominican Republic and is held in the Penney Research Collection, University of Manchester (number: PRC/DRA/Eph/002). La Bucara is close to the famous La Toca Group of mines, but has only been developed in the last few years. Dominican amber has recently been dated as approximately 16 million-years-old (Miocene: Burdigalian) [23], although some researchers expect the deposit may span from Miocene to Eocene (*ca*. 16–45 million years) in age as discussed by Poinar [24]; an overview of its geological setting, palaeontology and the palaeoenvironment can be found in Penney [1].

The specimen was scanned using the Xradia MicroXCT system at the Manchester X-ray Imaging Facility (MXIF), University of Manchester. Two scans comprising 1,200 and 2,100 projections were acquired at 50 keV with optical magnifications of 4x and 10x, resulting in a final pixel size of 5.2 and 1.7 µm respectively. In each case, phase-contrast was used to increase edge contrast, enabling the extraction of fine anatomical features for visualization and reconstruction (see also Movie S1). Further methodological details can be found in Penney et al. [25].

The light microphotographs were assembled from a stacked series of digital images recorded by a Nikon Coolpix 4500 camera mounted on a Leica M10 stereomicroscope with 0.63× and 1.6× planapochromatic objectives, following the combination technique described by Betz & Green [26]. The specimen was immersed in a small transparent container filled with non-reactive index matching fluid and covered with a colourless photographic filter. High resolution images with low depth of field were recorded at appropriate intervals and combined to produce a single high resolution and high depth of field image using the pyramid stack function of the freeware program CombineZP. The final image was checked for artifacts to ensure faithful representation of the object under study.

## Results and Discussion

In the fossil specimen considered here (Fig. 1), a beautifully preserved mature male imago of *Borinquena parva* (Leptophlebiidae: Atalophlebiinae) is associated with a tiny ?*Sphyrotheca* sp. (Sminthuridae) Collembola syninclusion. The springtail is situated resting along the basal anterior edge of the right forewing close to the costal brace–humeral plate [27] region, where it appears to have secured itself for transport using its prehensile antennae.

Not all assemblages and behaviours observed in amber are natural. Some result from stress behaviours during entrapment or from post-mortem movements of organisms due to the flowing resin, which may bring inclusions into close proximity with one another. Our reasoning for considering this a naturally occurring phoretic association (rather than a taphonomic artifact) is as follows: 1) the springtail is actually in contact with the mayfly, resting on the forewing at the costal brace (vein ScA+) and humeral plate region at the base of the wing, where the springtail was presumably sitting in the distinct v-shaped depression formed by the costa (C), subcosta (Sc) and radius anterior (RA) wing veins; this point would result in minimal chance of becoming dislodged during flight when compared to other potential attachment sites such as the legs or further along the wing; 2) phoresis in Collembola has already been documented in amber fossils, with springtails using their antennae as prehensile appendages to cling on to the host [9]; in our specimen the antennae are outstretched and oriented towards prominent ridge-like structures over which they were presumably hooked and from which the are separated by a distance of only 57.5 µm; 3) lack of taphonomic disarticulation or wing and tail bending in the mayfly is indicative of rapid demise and little post-mortem movement within the resin; 4) Collembola are among the most numerous terrestrial arthropods in wetland communities, with a small number of species living on the surface of water [28] and so it is not unreasonable to expect a phoretic association such as that observed in the fossil; 5) springtails in amber are often preserved in association with other springtails and with considerable particulate debris; however, syninclusions in this specimen consist of flying insects (e.g., Hymenoptera, Diptera, Coleoptera and Isoptera) with minimal particulate matter.

Our find represents the first record of phoresy in adult mayflies, fossil or extant. The lack of records of phoretic association in extant Epemeroptera species is perhaps due to the short lifespan of the adults and because their life cycle is predominantly associated around and over an aquatic environment with little chance for terrestrial hitch-hikers to climb on board. However, we expect that extant species no doubt act as hosts for transporting phoretic organisms, albeit rarely, and thus it has simply gone unnoticed to date. While phoresy is know from a single previous example in springtails, our finding represents the first example of a phoretic springtail using a winged insect for aerial dispersal.

A qualitative scale [29] for determining the reliability of observed behaviours in the fossil record ranges from 1 (unequivocal, usually reserved for the likes of mating insects in amber) to 7 (highly speculative, where the evidence is controversial at best). We believe the specimen described here is best placed in category 2A, because there is little doubt about the reliability of our interpretation although absolute certainty cannot be confirmed.

Given that many fossil springtails in Neotropical ambers are indistinguishable from extant forms [30], [31], we can expect that this behaviour is not restricted to extinct lineages, as was proposed as one possible conclusion regarding the phoretic association previously documented in Baltic amber [9]. The life-like fidelity resulting from rapid entombment and death of organisms in amber forming resins provides a unique snapshot of a prehistoric world [32], sometimes preserving behaviours that can also shed clues as to what to expect in the extant fauna, but which we may be unlikely to observe.

## Supporting Information

Movie S1 Rotating CT reconstruction of the mayfly and phoretic springtail, including a close-up ‘region of interest’ scan.
(WMV)
